# Sirtuin Family in Acute Kidney Injury: Insights into Cellular Mechanisms and Potential Targets for Treatment

**DOI:** 10.3390/biom15101445

**Published:** 2025-10-13

**Authors:** Songyuan Yang, Wu Chen, Siqi Li, Sheng Zhao, Fan Cheng

**Affiliations:** 1 Department of Urology, Renmin Hospital of Wuhan University, Wuhan 430060, China; 2 Department of Anesthesiology, Renmin Hospital of Wuhan University, Wuhan 430060, China

**Keywords:** AKI, SIRT, epigenetic modifications, targeted therapy, deacetylase, histone modifications

## Abstract

Acute kidney injury (AKI) is a frequent clinical and pathological condition, often resulting from factors like ischemia, toxins, or infections, which cause a sudden and severe decline in renal function. This, in turn, significantly affects patients’ overall health and quality of life. The Sirtuin family (SIRTs), a group of Nicotinamide Adenine Dinucleotide (NAD+)-dependent deacetylases, is critically involved in key biological processes such as cellular metabolism, stress responses, aging, and DNA repair. Recent research has highlighted the vital role of SIRTs, such as SIRT1, SIRT3, and SIRT6, in the development and progression of AKI. These proteins help mitigate renal injury and facilitate kidney repair through mechanisms like antioxidant activity, anti-inflammatory responses, cellular repair, and energy metabolism. Additionally, the deacetylase activity of the SIRTs confers protection against AKI by modulating mitochondrial function, decreasing oxidative stress, and regulating autophagy. Although the precise mechanisms underlying the role of Sirtuins in AKI are still being explored, their potential as therapeutic targets is increasingly being recognized. This paper will discuss the mechanisms by which the SIRTs influence AKI and examine their potential in a future therapeutic strategy.

## 1. Introduction

AKI is a critical and common clinical condition, typically characterized by elevated serum creatinine levels and/or reduced urine output [1,2,3]. The most prevalent causes of AKI are ischemia–reperfusion injury, drug toxicity, and infections [4,5,6]. AKI affects 10–15% of hospitalized patients worldwide and is independently associated with a significantly higher risk of mortality [7,8,9]. Although AKI is often considered a self-limiting condition, it has the potential to progress to chronic kidney disease (CKD) and, eventually, renal failure [10,11,12]. Currently, there is no specific treatment available for AKI, and management primarily relies on supportive therapies, such as fluid management, diuretics, and dialysis [13,14,15]. Therefore, comprehensive research into the underlying pathological mechanisms of AKI is essential for developing early diagnostic methods and innovative therapeutic strategies to improve patient outcomes.

SIRTs are a group of NAD+-dependent deacetylases that are widely distributed across eukaryotic organisms. These enzymes play a key role in several biological processes, such as regulating cellular metabolism, managing stress responses, controlling aging, and facilitating DNA repair, through the deacetylation and modulation of various target proteins [16,17,18]. The SIRTs comprise seven members (SIRT1-SIRT7), each of which is essential for critical physiological functions, including energy metabolism, antioxidation, anti-inflammatory responses, and the aging process [19,20,21]. Numerous studies have shown that Sirtuins are involved in the onset and progression of a variety of diseases, including metabolic disorders, cardiovascular diseases, and neurodegenerative conditions [22,23,24,25]. Additionally, recent research has pointed out that SIRTs play a vital role in the progression of AKI.

This review was conducted by searching the PubMed database for studies on the SIRTs in AKI and discusses the role of SIRTs in the regulation of AKI, focusing on aspects such as cellular energy homeostasis, DNA repair, and antioxidant defense. The analysis particularly highlights the SIRTs that have been most extensively studied: SIRT1, SIRT3, and SIRT6. Lastly, we underline the potential of SIRT as a therapeutic target to reduce AKI in humans, while also emphasizing the importance of developing novel strategies aimed at activating these proteins.

### An Overview of the Sirtuin Family

**Figure 1:**
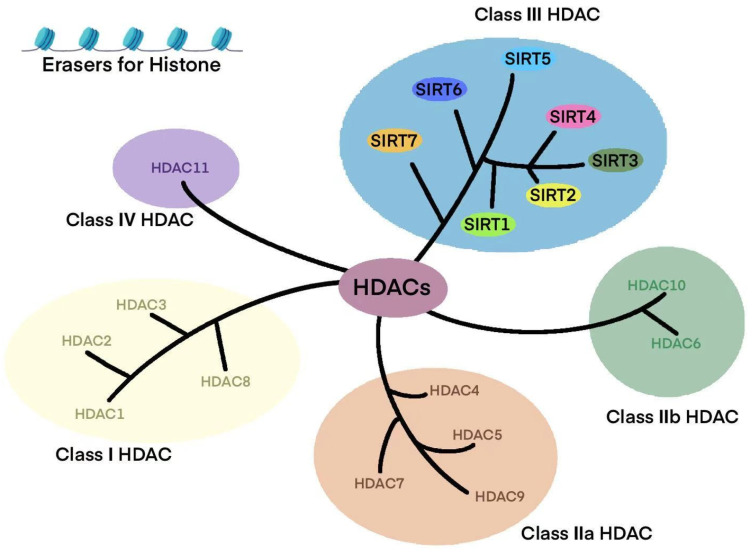
The classification of SIRTs. This diagram illustrates that SIRT1 and SIRT2 are mainly localized in the nucleus and cytoplasmhe classification and functions of histone deacetylases (HDACs). HDACs are enzymes that regulate gene expression by removing acetyl groups from histones. Based on their structure and function, HDACs are divided into several classes. Class I HDACs (HDAC1, HDAC2, HDAC3, and HDAC8) are primarily localized in the nucleus and play a key role in transcriptional regulation. Class IIa HDACs (HDAC4, HDAC5, HDAC7, and HDAC9) can shuttle between the nucleus and cytoplasm, influencing various cellular processes. Class IIb HDACs (HDAC6 and HDAC10) are mainly found in the cytoplasm, where they are involved in microtubule regulation and protein degradation. Class III HDACs, also known as sirtuins (SIRT1 to SIRT7), are NAD+-dependent enzymes that play critical roles in metabolism, aging, and stress responses. Class IV HDACs (HDAC11) represent a unique group with distinct enzymatic properties. HDAC: Histone Deacetylase; SIRT: Sirtuin.

SIRTs are NAD+-dependent deacetylases, originally identified in Saccharomyces cerevisiae, where they were named yeast silent information regulator 2 (Sir2) [26,27]. In mammals, these enzymes are known as SIRTs. SIRTs belong to the class III histone deacetylase (HDAC) family [28,29,30]. The total number of HDAC enzymes is 18, which are classified into four groups: class I (Rpd3-like proteins: HDAC1, HDAC2, HDAC3, HDAC8) [31,32]; class II (Hda1-like proteins: HDAC4, HDAC5, HDAC6, HDAC7, HDAC9, HDAC10) [33,34]; class III (Sir2-like proteins: SIRT1, SIRT2, SIRT3, SIRT4, SIRT5, SIRT6, SIRT7) [35,36]; and class IV (HDAC11) [37]. In mammals, the SIRT family is categorized under the class III histone deacetylase group, comprising seven members (SIRT1-SIRT7) (Figure 1).

**Figure 2:**
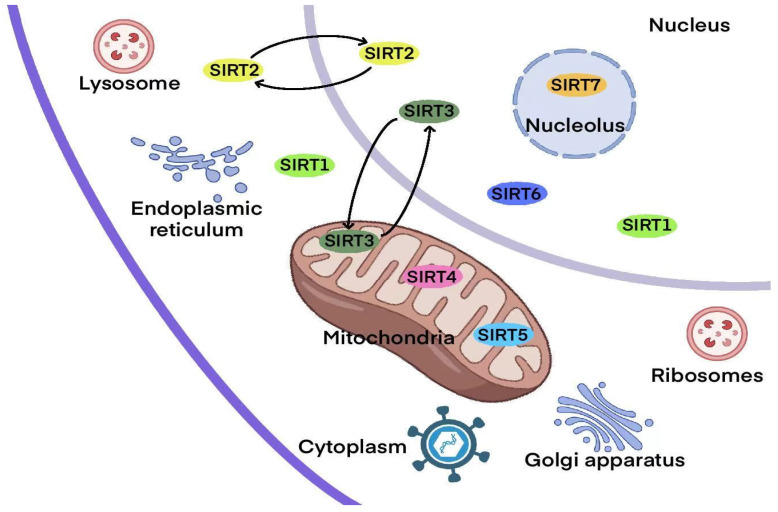
The subcellular localization of SIRTs. This diagram shows the distribution of different sirtuins (SIRTs) in various cellular compartments, highlighting their involvement in numerous biological processes. Sirtuins are NAD+-dependent deacetylases that play significant roles in regulating cellular metabolism, stress response, and aging. The chart displays where each sirtuin is localized: in the mitochondria (SIRT3, SIRT4, SIRT5), endoplasmic reticulum (SIRT1), cytoplasm (SIRT1, SIRT2), nucleus (SIRT6, SIRT7), nucleolus (SIRT7), and lysosome. This highlights the multifunctional and compartmentalized nature of sirtuins within the cell. SIRT: Sirtuin.

The distribution of SIRT family members differs, which affects their substrate specificity, interactions with intracellular regulatory molecules, and the functional diversity of SIRT enzymes. SIRT1 and SIRT2 are mainly localized in the nucleus and cytoplasm [38,39]. SIRT3, SIRT4, and SIRT5 are located in the mitochondria [40,41,42]. In contrast, SIRT6 and SIRT7 are predominantly found in the nucleus [43,44]. While SIRT1 is mostly confined to the nucleus, it can also be translocated to the cytoplasm. SIRT2, however, shuttles between the nucleus and cytoplasm, predominantly performing its function in the cytoplasm (Figure 2).

Acetylation is a crucial post-translational modification. The acetylation modification occurring on histones can affect gene expression by altering chromatin structure. On the other hand, acetylation modifications on non-histone proteins can regulate their activity or degradation. SIRTs are crucial epigenetic regulators that modulate various biological processes, including gene expression, cell cycle progression, and differentiation, through their deacetylation activity [45,46,47]. Deacetylation is involved in several critical biological processes, such as DNA repair, transcriptional regulation, lipid mobilization, apoptosis, and aging [48,49,50]. Additionally, SIRTs are involved in various vital functions, including cell proliferation, differentiation, adhesion, gene transcription, energy metabolism, stress response, inflammation regulation, and cancer development and metastasis [51,52,53].

Recent studies have highlighted the dual roles of SIRTs in diseases, where they can either promote disease progression or, at times, inhibit it. For example, in lung cancer research, it has been demonstrated that silencing SIRT1 regulates autophagy in non-small cell lung cancer cells with EML4-ALK L1196M and EML4-ALK G1202R mutations through the AMPK/mTOR/S6K signaling pathway, thereby promoting Epithelial–Mesenchymal Transition (EMT) and resistance to crizotinib [54]. This evidence deepens our understanding of the tumor-suppressive function of SIRT1 in lung cancer. On the other hand, other studies indicate that SIRT1 may contribute to the progression of non-small cell lung cancer via the Phosphoinositide 3-Kinase/Protein Kinase B (PI3K/AKT) signaling pathway [55]. These discrepancies in effects are mainly attributed to differences in the molecular pathways involved.

The functions of the SIRT family are not limited to deacetylation but also include various other de-modifying activities such as depropanoylation, demyristoylation, desuccinylation, and deglutamylation [16,56,57,58]. Among the family members, SIRT1, SIRT2, and SIRT3 show higher deacetylase activity, while SIRT4-7 demonstrate lower enzymatic activity in specific functions. SIRT3, which serves as the primary mitochondrial deacetylase, plays a pivotal role in regulating both mitochondrial function and oxidative stress. Furthermore, SIRT6 is crucial for DNA repair and maintaining chromatin stability (Table 1).

In summary, SIRTs regulate numerous biological processes through activities such as deacetylation, depropanoylation, and other modifications, impacting cell metabolism, chromatin remodeling, RNA transcription, inflammation, cell cycle regulation, programmed cell death, immune responses, and the management of oxidative stress. As essential biological regulators, SIRTs have become promising targets for treating a wide range of diseases, including cancer, metabolic disorders, and aging, demonstrating considerable potential for clinical application.

## 2. Mechanisms of the Sirtuin Family in AKI

Research on SIRT1 in AKI has been extensive since 2010, with studies showing that overexpressing SIRT1 in the kidneys significantly reduces cisplatin-induced AKI [65], and pharmacological activation of SIRT1 also mitigates cisplatin-induced AKI [66]. In 2013, a study further confirmed the protective role of SIRT1 in AKI [67]. This research is particularly noteworthy, as it found that young mice had higher SIRT1 levels and demonstrated milder ischemia–reperfusion-induced AKI. When SIRT1 was knocked down, kidney injury worsened, suggesting that SIRT1 may regulate the protective effect in younger mice against AKI. Subsequent studies have also affirmed the protective role of SIRT1 in other AKI models [68]. However, these studies did not explore the underlying mechanisms. Following this, research began to focus on how SIRT1 regulates AKI. For instance, the Gao Q team showed that SIRT1 alleviates lipopolysaccharide (LPS)-induced AKI by inhibiting the activation of the NLRP3 inflammasome in the nucleotide-binding oligomerization domain-like receptor (NLR) family pyrin domain [69]. However, they did not examine the downstream effects of SIRT1. Research by Sun M and colleagues identified the downstream effects of SIRT1, showing that SIRT1 mitigates sepsis-induced AKI by promoting p53 deacetylation, thereby enhancing autophagy [70]. Other studies have demonstrated that drugs such as baicalein or astaxanthin can alleviate polymyxin or contrast-induced AKI by activating SIRT1 and promoting p53 [71,72]. Furthermore, studies have shown that SIRT1 deacetylates and regulates p53, inhibiting apoptosis and thus alleviating AKI [73]. Similarly, the Wei S et al. focused on SIRT1′s regulation of non-histone deacetylation to alleviate AKI. They found that SIRT1-mediated HMGB1 deacetylation inhibits sepsis-associated AKI [74]. However, they did not investigate how HMGB1′s downstream effects regulate kidney injury.

SIRT2 shares a similar intracellular distribution to SIRT1, but studies on its role in AKI are still limited. One study demonstrated that disrupting SIRT2′s enzymatic activity and its ability to deacetylate Forkhead Box O3a (FOXO3a) resulted in increased expression of Bim and caspase3, thus aggravating renal ischemia/reperfusion injury [75].

SIRT3 plays a key role in regulating mitochondrial function due to its presence in the mitochondria. For example, Morigi M was the first to identify that SIRT3 alleviates kidney injury by enhancing mitochondrial dynamics [76]. They confirmed the protective effect of SIRT3 in AKI, and numerous subsequent studies have further supported its role in AKI [77,78], although the underlying mechanism of SIRT3′s action remains unexplored. The Zhao W was the first to uncover the regulatory mechanism behind SIRT3′s protective effect in AKI. They discovered that SIRT3 protects against AKI by modulating autophagy through the AMP-activated protein kinase/mechanistic target of rapamycin (AMPK/mTOR) pathway [79]. However, the specific mechanism by which SIRT3 regulates the AMPK pathway was not elucidated. Research has shown that SIRT3 alleviates AKI by inhibiting p53 acetylation [80] thereby regulating p53 acetylation to mediate its effects. However, the role of p53 in regulating kidney injury was not examined. The Jian Y et al. demonstrated that SIRT3 promotes Optic Atrophy 1 (OPA1)-mediated mitochondrial fusion by deacetylating Yme1-like 1 (YME1L1), reducing LPS-induced mitochondrial damage in renal tubular epithelial cells [81]. Additionally, activation of the SIRT3/OPA1 pathway mitigates mitochondrial dysfunction and inflammation, thus alleviating cisplatin-induced AKI [82]. Moreover, studies suggest that SIRT3 may regulate fatty acid oxidation (FAO) by deacetylating liver kinase B1 and activating AMP-activated protein kinase, which could alleviate AKI [83].

Research on SIRT4 and SIRT5 in AKI is still limited, with studies indicating that SIRT5 may mitigate AKI [84,85,86], possibly by regulating the Nuclear factor erythroid 2-related factor 2/Heme oxygenase 1 (Nrf2/HO-1) pathway [86].

SIRT6 is predominantly found in the nucleus, where it influences gene transcription. Zhang Y was the first to confirm the protective role of SIRT6 in AKI [87]. Pharmacological activation of SIRT6 has also been shown to alleviate AKI [88]. Studies have highlighted that SIRT6 mitigates AKI by inhibiting the Amyloid β-Composite Proteinase, Secondary Structure Domain (ACMSD) signaling pathway, enhancing lipid metabolism, and reducing apoptosis in renal tubular epithelial cells [89]. However, these studies did not investigate how SIRT6 regulates the expression of ACMSD or whether its effect is dependent on histone deacetylase activity. Our team conducted in-depth research and discovered that SIRT6 protects against AKI by reducing acetylation at histone H4K9ac, inhibiting BRCA1-associated protein 1 (BAP1) expression, and suppressing ferroptosis [5].

The role of SIRT7 in AKI has been the subject of ongoing debate. Several studies suggest that the deletion or degradation of SIRT7 may reduce the expression of tumor necrosis factor-α (TNF-α) by influencing the nuclear translocation of the transcription factor nuclear factor kappa B, which in turn mitigates the effects of cisplatin-induced AKI [90,91,92]. Furthermore, research indicates that inhibiting SIRT7 activity in the kidneys of septic rats can suppress pyroptosis and related protein expression, further alleviating AKI [93]. In contrast, Wang Y reported that silencing SIRT7 led to apoptosis and kidney dysfunction associated with renal ischemia/reperfusion injury [94]. These divergent findings seem unconvincing if solely attributed to variations in experimental models. Moreover, Wang Y also observed that silencing SIRT7 resulted in an increase in FOXO3a, Bim, and caspase3 levels, a finding similar to their own study on SIRT2, which raises substantial concerns regarding the validity of their conclusions [75].

To conclude, SIRTs play a key protective role in AKI, with SIRT1, SIRT2, SIRT3, SIRT5, and SIRT6 each exerting their protective effects via distinct mechanisms. In contrast, SIRT7 has been shown to have a detrimental role, and the underlying reasons for this difference are an important focus for future research. Furthermore, there is a notable lack of research on SIRT4 in AKI, which suggests that it should be a priority for future studies (Table 2).

## 3. The Role of the Sirtuin Family in AKI Treatment

### 3.1. Traditional Chinese Medicine or Natural Compounds Targeting SIRTs in the Treatment of AKI

Traditional Chinese medicine (TCM) and natural compounds have gained wide application in the treatment of various diseases, including cancer, hypertension, and coronary heart disease, due to their multitarget effects, integrated regulatory mechanisms, fewer side effects, and better long-term efficacy [95,96,97]. Recent studies have demonstrated that many of these medicines and compounds protect the kidneys by specifically targeting the SIRTs. For example, a study by Qiongyue Z et al. revealed that irisgenin mitigates AKI in septic mice by inhibiting ferroptosis via the SIRT1/Nrf2 pathway [68]. In a similar fashion, Tang J et al. identified that Agrimol B alleviates cisplatin-induced AKI by activating the SIRT1/Nrf2 signaling pathway in mice [98]. Furthermore, research by Qiu CW showed that Gastrodin suppresses ferroptosis through the SIRT1/FOXO3A/Glutathione Peroxidase 4 (GPX4) pathway, thereby reducing cisplatin-induced nephrotoxicity [99]. These findings underscore the essential role of SIRT1 in regulating ferroptosis, although variations in experimental models may lead to differences in the downstream pathways observed.

In addition to ferroptosis and oxidative stress, certain ingredients from Chinese medicine also exhibit the ability to inhibit other forms of programmed cell death by activating SIRT. For instance, Zha C and colleagues discovered that intravenous administration of Astragaloside IV targets the SIRT1/FOXO3a axis to prevent AKI [100]. Similarly, studies have demonstrated that astaxanthin can inhibit apoptosis induced by damaging agents via the activation of the SIRT1/FOXO3a pathway [101]. Moreover, compounds such as ginkgolide B [102], rutin [103], daidzein [104], and flavonoids (TF) [105] are able to alleviate AKI by activating SIRT1. Additionally, silymarin [106] and glycyrrhizin [107] have been shown to help mitigate AKI by activating SIRT3.

### 3.2. Nanoparticles Targeting SIRTs in the Treatment of AKI

Nanoparticles have demonstrated considerable therapeutic efficacy by enabling the targeted delivery of drugs, enhancing their bioavailability, and minimizing side effects [108]. Research indicates that the development of ultra-small polyphenol-NAD nanoparticles can restore kidney function, stabilize the immune microenvironment, and maintain mitochondrial function in AKI mice via the NAD-Sirtuin-1 axis, effectively alleviating or even preventing AKI [109]. Additionally, porous Se@SiO_2_ nanoparticles have been found to alleviate cisplatin-induced AKI through the activation of SIRT1 [110].

### 3.3. Clinical Drugs Targeting SIRTs in the Treatment of AKI

Several clinical drugs have also been proven to protect renal function by targeting the SIRT pathway. For instance, Zhang S discovered that dexmedetomidine (DEX), an α2 adrenergic receptor (α2-AR) agonist, protects mitochondrial integrity, reduces oxidative stress, prevents cell death, and mitigates septic AKI by upregulating the α2-AR/SIRT1/Peroxisome proliferator-activated receptor gamma coactivator 1-alpha (PGC-1α) pathway [111]. Furthermore, research has demonstrated that the aldosterone receptor antagonist eplerenone mitigates renal ischemia/reperfusion injury through the modulation of the SIRT1/SIRT3/PGC-1α signaling pathway [112].

Canagliflozin (Cana), a sodium-glucose cotransporter 2 (SGLT-2) inhibitor, is commonly used in the clinical management of diabetes. Other studies suggest that canagliflozin can activate the SIRT1/FOXO-3a/PGC-1α pathway, thereby promoting renal protection against glycerol-induced AKI [113]. Likewise, dapagliflozin, another SGLT-2 inhibitor, can reduce AKI in diabetic patients by stimulating the SIRT3/PGC1-α signaling pathway, which helps mitigate abnormal metabolic reprogramming [114].

Certain hormones have been shown to alleviate AKI by targeting SIRT1. For example, research has demonstrated that melatonin can prevent AKI in rats with severe burns through the activation of SIRT1 [115]. Additionally, melatonin regulates abnormal autophagy and ferroptosis by SIRT1-mediated deacetylation of p53, which reduces AKI caused by ischemia–reperfusion [116]. Moreover, melatonin mitigates acute renal ischemia/reperfusion injury in diabetic rats by activating the SIRT1/Nrf2/HO-1 signaling pathway [117]. Beyond activating SIRT1, studies have also confirmed that melatonin promotes mitochondrial autophagy through SIRT3-mediated deacetylation of TFAM, thereby alleviating AKI induced by sepsis [118]. Furthermore, melatonin supports mitochondrial stability and integrity through the activation of SIRT3/SOD2, which reduces renal ischemia–reperfusion injury [119]. This clearly underscores the therapeutic potential of melatonin in AKI via the activation of SIRT1. Moreover, erythropoietin promotes energy metabolism and reduces cellular damage through the SIRT1/PGC1-α pathway [120].

### 3.4. Stem Cell Therapy Targeting SIRTs in the Treatment of AKI

Stem cell therapy involves introducing bone marrow-derived mesenchymal stem cells into the body. Research has shown that these cells promote tissue repair, decrease inflammation, and reduce fibrosis, leading to improved therapeutic outcomes in AKI [121]. This effect is primarily mediated by the upregulation of the SIRT1/Parkin pathway, which inhibits apoptosis and ferroptosis in renal tubular epithelial cells (RTECs) within the kidney tissue. Alongside stem cell therapy, exosome therapy employs small vesicles (exosomes) secreted by cells to transport bioactive molecules, such as proteins and RNA, to damaged tissues, thereby facilitating tissue repair and regeneration. Studies have indicated that exosomes derived from adipose tissue mesenchymal stem cells exert protective effects in AKI through the SIRT1 pathway [122].

In conclusion, the SIRTs, particularly SIRT1 and SIRT3 (Table 3), has shown substantial therapeutic promise in basic research focused on targeting SIRTs for the treatment of AKI. By incorporating TCM components, alongside certain clinical drugs used for other conditions, or even utilizing exosomes and nanoparticles designed to activate SIRT1 or SIRT3, AKI can be significantly alleviated. Additionally, melatonin has been thoroughly substantiated as a protective agent against AKI, operating through multiple mechanisms, and has emerged as a promising candidate for clinical applications in the future.

## 4. Summary and Outlook

AKI is a common and serious clinical condition. Although current treatment options primarily rely on supportive care, such as fluid therapy and dialysis, their clinical effectiveness remains limited. Recently, the SIRTs, which consist of key NAD+-dependent deacetylases, have been increasingly recognized for their crucial involvement in the initiation and progression of AKI. Research has shown that SIRT members, such as SIRT1 and SIRT3, offer significant protection to the kidneys by modulating oxidative stress, inflammation, cellular repair, and energy metabolism. The recognized protective effects of SIRT1 and SIRT3 in AKI have highlighted the SIRTs as a promising therapeutic target.

As research into the mechanisms of the SIRTs in AKI progresses, an increasing variety of natural and clinical drugs has been identified that enhance kidney function through the activation of SIRT1 or SIRT3. For example, natural compounds such as Astragaloside IV, astaxanthin, and clinical agents like melatonin, dexmedetomidine, and canagliflozin have been shown to exert protective effects by targeting the SIRT signaling pathway. In addition, stem cell therapy and exosome therapy have demonstrated considerable potential in mediating their effects via the regulation of SIRTs.

Although there have been advancements in research on the SIRTs in AKI, many mechanisms remain poorly understood, and the roles of various SIRT family members in different types of AKI still require further investigation. In particular, research on SIRT4 in AKI is limited, while findings concerning SIRT7 are still controversial. Future studies should prioritize clarifying the specific mechanisms of action for each SIRT family member in AKI and developing more selective drugs or treatment approaches to optimize their clinical efficacy.

In this process, appropriate clinical study design is crucial. Future research could consider different types of clinical studies, such as randomized controlled trials (RCTs), longitudinal observational cohort studies, and biomarker-based prognostic studies. These studies will help determine whether Sirtuin modulation can effectively improve the management of AKI and assess its potential in renal function recovery or chronic kidney disease (CKD) progression. In particular, randomized controlled trials may be the most informative approach to validate the efficacy and safety of different Sirtuin modulation strategies in AKI patients.

In conclusion, the SIRTs, particularly SIRT1 and SIRT3, have demonstrated considerable potential in treating AKI. Future treatments are expected to incorporate a combination of TCM, clinical drugs, stem cell therapies, exosomes, and nanoparticles, offering safer and more effective therapeutic alternatives for AKI patients. As both technological advancements and research progress, therapies targeting the SIRTs are likely to become increasingly important in the clinical management of AKI.

## Figures and Tables

**Table 1:** Classification, function and characteristics of mammal SIRTs.

SIRT	Class	Cellular Localization	Enzymatic Activity	Histone Deacetylation Target	Biological Function	Refs.
SIRT1	I	Nucleus and cytoplasm	Deacetylase, Depropionylase	H1-K26Ac, H3-K9Ac, H4-K16Ac	Chromatin modification, DNA repair, cell cycle regulation, cell metabolism and survival	[59]
SIRT2	I	Nucleus and cytoplasm	Deacetylase, Demyristoylase, Depropionylase	H3-K18Ac, H3-K56Ac, H4-K16Ac	Cell cycle regulation, microtubule dynamics, inflammation, differentiation	[60]
SIRT3	I	Mitochondria	Deacetylase, Depropionylase	H3-K56Ac, H4-K14Ac	Apoptosis, nuclear gene expression, control of metabolism	[27]
SIRT4	II	Mitochondria	Deacetylase, ADP ribosyltransferase, Biotinidase, Lipoamidase	H4-K16Ac	Resistance, genomic stability, energy metabolism	[61]
SIRT5	III	Mitochondria	Deacetylase, Demethylase, Desuccinylase, Glutaminase	Unknown	Mitochondrial metabolism, amino acid degradation, cellular respiration, reactive oxygen species management	[62]
SIRT6	IV	Nucleus	Deacetylase, ADP-ribosylation, Defattyacylation	H3-K9Ac, H3-K18Ac, H3-K56Ac	Cell proliferation, energy metabolism, DNA damage repair, stem cell differentiation	[63]
SIRT7	IV	Nucleolus	Deacetylase, Desuccinylase	H3-K18Ac	DNA repair, RNA transcription, metabolism regulation	[64]

SIRT1: Sirtuin 1; SIRT2: Sirtuin 2; SIRT3: Sirtuin 3; SIRT4: Sirtuin 4; SIRT5: Sirtuin 5; SIRT6: Sirtuin 6; SIRT7: Sirtuin 7; H1-K26Ac: Histone H1 at lysine 26 acetylation; H3-K9Ac: Histone H3 at lysine 9 acetylation; H4-K16Ac: Histone H4 at lysine 16 acetylation; H3-K18Ac: Histone H3 at lysine 18 acetylation; H3-K56Ac: Histone H3 at lysine 56 acetylation; ADP: Adenosine diphosphate; RNA: Ribonucleic acid.

**Table 2:** The roles of SIRTs in AKI, their targets, and the mechanisms that drive their effects.

SIRT	Protection/Damage	Disease Model	Active Molecule	Mechanism of Action	Reference
SIRT1	Protection	Cisplatin-induced AKI, ischemia–reperfusion AKI, sepsis-induced AKI	Unclear	Unclear	[66,67,68]
	Protection	Sepsis-induced AKI	Unclear	Overexpression of Sirtuin 1 (SIRT1) alleviates lipopolysaccharide (LPS)-induced AKI by inhibiting the activation of the nucleotide-binding oligomerization domain-like receptor (NLR) family pyrin domain-containing 3 (NLRP3) inflammasome.	[69]
	Protection	Sepsis-induced AKI	P53	SIRT1 promotes deacetylation of p53, thereby promoting autophagy to alleviate AKI caused by sepsis.	[70]
	Protection	Cisplatin-induced AKI	P53	The SIRT1/P53/BAX pathway alleviates cisplatin-induced apoptosis.	[73]
	Protection	Sepsis-induced AKI	HMGB1	SIRT1-mediated deacetylation of HMGB1 inhibits sepsis-associated AKI.	[74]
SIRT2	Protection	Ischemia–reperfusion AKI	FOXO3a	SIRT2 deacetylates FOXO3a and inhibits cell apoptosis.	[75]
SIRT3	Protection	Cisplatin-induced AKI	Unclear	SIRT3 can improve kidney injury by improving mitochondrial dynamics.	[76]
	Protection	Sepsis-induced AKI	Unclear	SIRT3 prevents AKI through autophagy regulated by AMPK/mTOR.	[79]
	Protection	Ischemia–reperfusion AKI	P53	SIRT3 alleviates AKI by inhibiting the acetylation of p53.	[80]
	Protection	Sepsis-induced AKI	YME1L1	SIRT3 promotes OPA1-mediated mitochondrial fusion by deacetylating YME1L1, alleviating mitochondrial damage induced by LPS in renal tubular epithelial cells.	[81]
	Protection	Cisplatin-induced AKI	Hepatic kinase B1	SIRT3 may regulate FAO by deacetylating hepatic kinase B1 and activating AMP-activated protein kinase, thereby alleviating AKI.	[83]
SIRT5	Protection	Cisplatin-induced AKI	Unclear	SIRT5 alleviates AKI by regulating Nrf2/HO-1.	[86]
SIRT6	Protection	Sepsis-induced AKI	Unclear	SIRT6 alleviates AKI by inhibiting the ACMSD signaling pathway, enhancing lipid metabolism, and reducing renal tubular epithelial cell apoptosis.	[89]
	Protection	Cisplatin-induced AKI	H4K9ac	SIRT6 alleviates ferroptosis in cisplatin-induced AKI by inhibiting the BAP1/xCT signaling axis.	[5]
SIRT7	Damage	Cisplatin-induced AKI	Unclear	Loss of SIRT7 reduces tumor necrosis factor-alpha (TNF-α) expression by regulating the nuclear expression of the transcription factor NF-kB.	[90,91]
SIRT7	Protection	Ischemia–reperfusion AKI	Unclear	Silencing of SIRT7 leads to cell apoptosis and renal dysfunction caused by renal ischemia/reperfusion injury.	[94]

SIRT1: Sirtuin 1; AKI: Acute Kidney Injury; LPS: Lipopolysaccharide; NLRP3: Nucleotide-binding oligomerization domain-like receptor family pyrin domain-containing 3; FOXO3a: Forkhead box O3a; AMPK: AMP-activated protein kinase; mTOR: Mechanistic target of rapamycin; YME1L1: Yme1-like 1; OPA1: Optic Atrophy 1; FAO: Fatty Acid Oxidation; Nrf2: Nuclear factor erythroid 2-related factor 2; HO-1: Heme oxygenase 1; ACMSD: Amyloid β-Composite Proteinase, Secondary Structure Domain; BAP1: BRCA1-associated protein 1; H4K9ac: Histone H4 at lysine 9 acetylation; TNF-α: Tumor necrosis factor-alpha; NF-kB: Nuclear factor kappa-light-chain-enhancer of activated B cells.

**Table 3:** Summary of pharmacological agents and bioactive compounds modulating sirtuin pathways and their potential nephroprotective effects.

Drug/Compound	Sirtuin Pathway	Mechanism of Action	Potential Nephroprotective Effect	Reference
Irisin	SIRT1/Nrf2	Activates SIRT1/Nrf2 pathway to inhibit ferroptosis	Alleviates AKI in septic mice	[98]
Agrimol B	SIRT1/Nrf2	Activates SIRT1/Nrf2 signaling pathway	Relieves cisplatin-induced AKI	[99]
Gastrodin	SIRT1/FOXO3A/GPX4	Inhibits ferroptosis via SIRT1/FOXO3A/GPX4 pathway	Reduces cisplatin nephrotoxicity	[100]
Astragaloside IV	SIRT1/FOXO3A	Intravenous injection targeting SIRT1/FOXO3a axis to suppress pyroptosis	Inhibits AKI pyroptosis	[101]
Astaxanthin	SIRT1/FOXO3A	Activates SIRT1/FOXO3a pathway, inhibits apoptosis	Alleviates drug-induced apoptosis	[102]
Ginkgolide/Rutin/Isoflavone/Flavonoids	SIRT1	Activates SIRT1	Relieves AKI	[103,104,105]
Silymarin	SIRT1/SIRT3	Activates SIRT3	Relieves AKI	[106,107]
Glycyrrhizin	SIRT3	Activates SIRT3	Relieves AKI	[108]
Ultrasmall Polyphenol-NAD Nanoparticles	SIRT1	Restores AKI mouse kidney function and immune microenvironment via NAD-Sirtuin-1 axis	Effectively alleviates or prevents AKI	[109]
Porous Se@SiO_2_ Nanoballs	SIRT1	Activates SIRT1	Reduces cisplatin-induced AKI	[110]
Dexmedetomidine (DEX)	SIRT1	Upregulates α2-AR/SIRT1/PGC-1α pathway, protecting mitochondria structure and function	Reduces septic AKI	[111]
Eplerenone	SIRT1/SIRT3/PGC-1α	Regulates SIRT1/SIRT3/PGC-1α signaling pathway	Reduces renal ischemia/reperfusion injury	[112]
Canagliflozin (Cana)	SIRT1/FOXO-3a/PGC-1α	Activates SIRT1/FOXO-3a/PGC-1α pathway	Promotes kidney protection from glycerol-induced AKI	[113]
Dapagliflozin	SIRT3/PGC-1α	Activates SIRT3/PGC1-α signaling, reduces metabolic reprogramming	Alleviates AKI in diabetic patients	[114]
Melatonin	SIRT1	Activates SIRT1, regulating p53 deacetylation, autophagy, and pyroptosis	Prevents severe burn-induced AKI in rats	[115]
Melatonin	SIRT1/Nrf2/HO-1	Through SIRT1/Nrf2/HO-1 pathway, alleviates diabetic rat acute kidney ischemia/reperfusion injury	Reduces AKI	[116]
Melatonin	SIRT3	Mediates TFAM deacetylation via SIRT3, promotes mitochondrial autophagy	Alleviates sepsis-induced AKI	[117]
Melatonin	SIRT3/SOD2	Activates SIRT3/SOD2, maintaining mitochondrial stability and integrity	Reduces renal ischemia/reperfusion injury	[118]
Erythropoietin	SIRT1/PGC1-α	Promotes energy metabolism via SIRT1/PGC1-α pathway	Improves cell injury	[119]

## Data Availability

All data presented in this study are included within the paper.
